# Population structuring of the invasive mosquito *Aedes albopictus* (Diptera: Culicidae) on a microgeographic scale

**DOI:** 10.1371/journal.pone.0220773

**Published:** 2019-08-02

**Authors:** Laura Cristina Multini, Ana Letícia da Silva de Souza, Mauro Toledo Marrelli, André Barretto Bruno Wilke

**Affiliations:** 1 Department of Epidemiology, Faculty of Public Health, University of São Paulo, São Paulo, SP, Brazil; 2 São Paulo Institute of Tropical Medicine, University of São Paulo, São Paulo, SP, Brazil; 3 Department of Public Health Sciences, Miller School of Medicine, University of Miami, Miami, FL, United States of America; Faculty of Science, Ain Shams University (ASU), EGYPT

## Abstract

*Aedes albopictus* is an invasive mosquito species that has spread globally and can transmit several arboviruses, including dengue, chikungunya and yellow fever. The species was first reported in Brazil in 1986 and since then has been found in 24 of the 27 Brazilian states, often in peri-urban environments close to highly urbanized areas. To date, population genetics of this important mosquito in areas in the city of São Paulo has not been investigated. In this study, we used 12 microsatellite loci to investigate the microgeographic population genetics of *Ae*. *albopictus*, which is present throughout the city of São Paulo. All the analyses revealed structuring of the populations studied, divided into two groups with restricted gene flow between them and without evidence of isolation by distance. We propose two hypotheses to explain the results: (i) low dispersal capability—limited gene flow between populations is due to the low dispersal capability inherent to *Ae*. *albopictus*; and (ii) multiple introductions—the structure identified here results from multiple introductions, which led to different dispersal patterns within the city and more genetic heterogeneity. The ability of *Ae*. *albopictus* to invade new areas and expand may explain why these mosquito populations appear to be well established and thriving in the city of São Paulo.

## Introduction

There are currently more humans living in urban areas than ever in the history of civilization [1]. As a consequence of this escalating urbanization, significant environmental changes are taking place, resulting, among other things, in increasing temperatures and pollution of the natural environment [2,3].

Inhabiting urban areas is challenging for most species. However, invasive species are more likely to thrive in such environments than native species [2]. Furthermore, urbanization processes often result in a reduction in overall biodiversity followed by an increase in the abundance of the invasive species that have adapted to the urban environment [2,4]. This phenomenon is commonly observed in epidemiologically important mosquito species [4–6].

Mosquito species that have adapted to urban environments can not only be a source of nuisance but also transmit diseases, as in the case of *Aedes* (*Stegomyia*) *albopictus* (Skuse), an aggressive species well adapted to urban environments. This mosquito can breed in artificial containers (e.g., tires and cemetery urns) and blood feed on human hosts, completing its entire life cycle in urban environments [7–9].

*Aedes albopictus* is native to Asia and has spread to all continents except Antarctica, most likely because of its close relationship with humans [10,11]. As this mosquito undergoes diapause, its eggs can survive for months in artificial breeding containers until the environmental conditions are suitable for hatching and its life cycle can begin [12,13]. This has allowed *Ae*. *albopictus* to colonize new areas by being inadvertently dispersed in used tires imported from other countries [14,15]. The species’ adaptability to temperate climates [16–18] and resistance to several insecticides have also favored colonization [19–21].

*Aedes albopictus* was established in Brazil in 1986 and since then has spread to most of the country, where it is often found in suburban areas with higher vegetation coverage [22,23]. However, a recent entomological survey demonstrated initial evidence of *Ae*. *albopictus* domiciliation in a densely populated slum in Rio de Janeiro. The presence of the species in such an environment could indicate its increased adaptation to areas undergoing anthropogenic changes in Brazil [24].

*Aedes albopictus* is a competent vector for several arboviruses, including dengue (DENV), chikungunya (CHIKV), Zika (ZIKV) and yellow fever (YFV) [25–30], and was implicated as a primary vector for the CHIKV outbreak in Italy in which 205 cases were notified between July and September 2007 and one death was reported [31].

Even though the species is not implicated in dengue transmission in Brazil, the DENV-1 serotype has been isolated from its larvae [32]. In addition, ZIKV RNA fragments were found in field-collected eggs of *Ae*. *albopictus* in the state of Bahia, Brazil, in an active ZIKV transmission zone [27]. It is also possible that the species is acting as a bridge vector between wild and urban YFV transmission cycles in Brazil, as specimens infected with YFV were recently found in yellow-fever hotspots in two municipalities in the state of Minas Gerais, Brazil [33,34].

Urbanization processes are often responsible for driving the genetic diversity within urban populations of mosquitoes [2,6]. This phenomenon is undoubtedly occurring in mosquito species such as *Aedes aegypti*, *Aedes fluviatilis*, *Culex nigripalpus*, and *Culex quinquefasciatus* in the city of São Paulo, Brazil [35–38]. São Paulo is a megacity with approximately 12 million people in the center of a metropolitan area with a population of more than 20 million [39]. Thus, identifying the genetic structure of the exotic mosquito *Ae*. *albopictus* in São Paulo could lead to a better understanding of how anthropogenic changes to the environment in developing countries such as Brazil are modulating the population structure of this species and the implications of this for vector-borne disease transmission patterns [36,40].

Population genetic studies of *Ae*. *albopictus* have been carried out as this species continues to spread worldwide [8,40–43]. A population-structuring study of *Ae*. *albopictus* in Manaus, Brazil, found that populations of this species have undergone a recent expansion following a founder effect [44]. Moreover, studies of populations of the species in cities where it has recently become established have shown weak genetic structure and high gene flow, suggesting a dispersal pattern driven mostly by human movements [40,45].

Microsatellite markers are highly polymorphic genetic markers widely used in mosquito population genetics studies [46–48]. Previous studies using these markers provide valuable information on the microgeographic genetic structure of vector mosquitoes in São Paulo, Brazil [35–37]. Considering the high abundance and widespread presence of the invasive species *Ae*. *albopictus* in São Paulo [5], we hypothesize that it is successfully thriving in urban and peri-urban areas. The primary objective of this study was therefore to investigate the microgeographic population genetics of *Ae*. *albopictus* throughout the city of São Paulo.

## Material and methods

### Specimen collection

**Fig 1:**
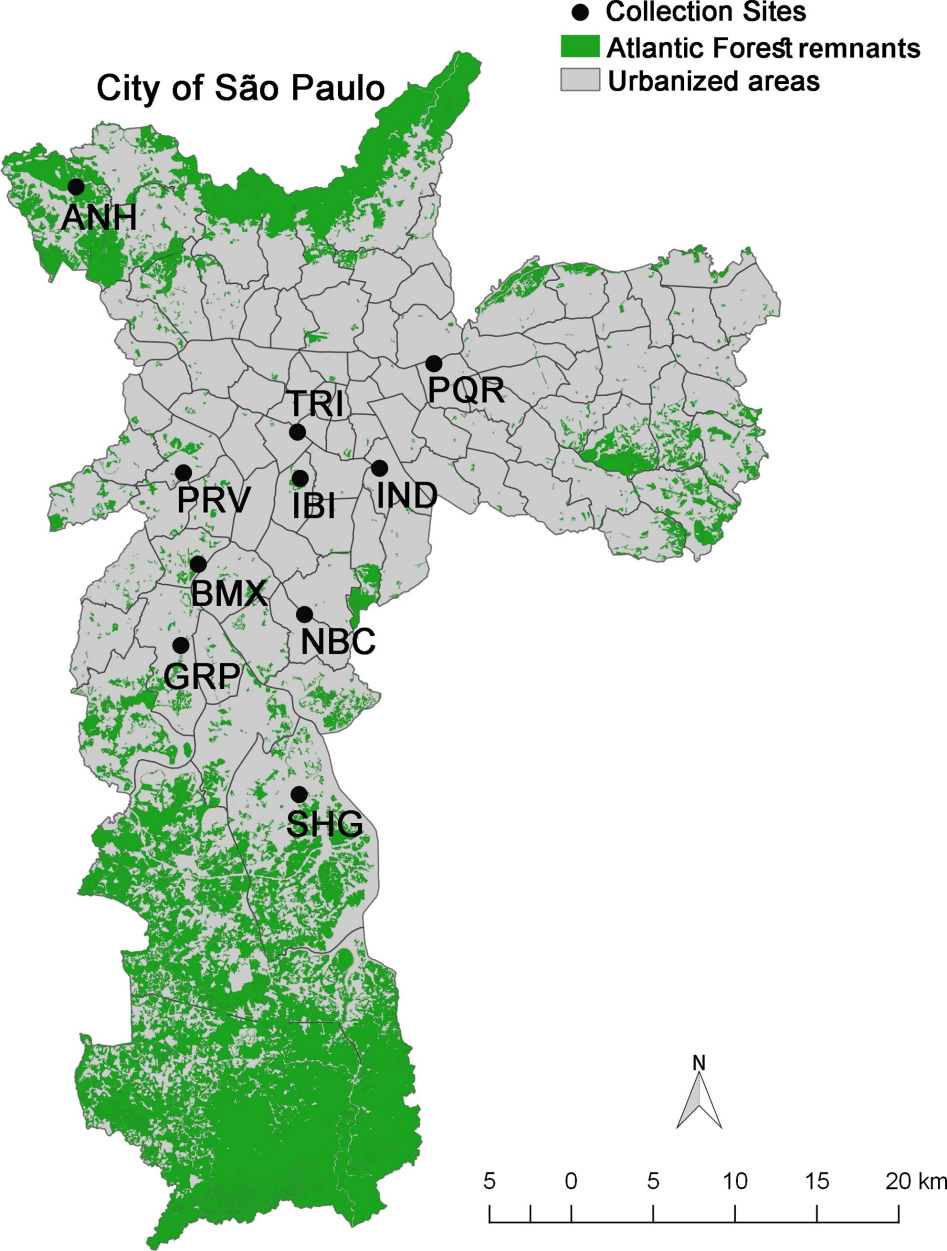
Map of the subdistricts of the city of São Paulo, Brazil, showing the parks where *Aedes albopictus* specimens were collected. Anhanguera (ANH), Burle Marx (BMX), Piqueri (PQR), Trianon (TRI), Guarapiranga (GRP), Ibirapuera (IBI), Independência (IND), Previdência (PRV), Shangrilá (SHG) and Nabuco (NBC). The map was created with QGIS v2.18.9 (http://www.qgis.org) using layers freely available at http://geosampa.prefeitura.sp.gov.br/PaginasPublicas/_SBC.aspx#. The layers used to the construction of the map were “Administrative limits–District” and “Green Natural Resources–PMMA”.

*Aedes albopictus* samples were collected from 10 urban parks (Anhanguera, Piqueri, Trianon, Burle Marx, Guarapiranga, Ibirapuera, Previdência, Shangrilá, Independência and Nabuco) in different areas of the city of São Paulo (Table 1) [5,49,50]. The parks are relatively small green urban spaces within highly urbanized areas of the city [5] and were chosen because of the need for collection sites in urbanized areas with high population densities where the traps were unlikely to be tampered with and the researchers would not be exposed to any dangers (Fig 1).

**Table 1:** *Aedes albopictus* sampling information.

Collection Site	Coordinates	N	Year
ANH	23°25'12.42"S 46°46'55.39"W	30	2012
BMX	23°37'54.06"S 46°43'16.81"W	19	2013
PQR	23°31'41.23"S 46°34'26.65"W	30	2013
TRI	23°33'42.53"S 46°39'27.96"W	15	2011
GRP	23°40'33.10"S 46°44'3.71"W	30	2011
IBI	23°35'14.70"S 46°39'27.48"W	29	2012
IND	23°35'2.99"S 46°36'35.38"W	30	2011
PRV	23°34'51.09"S 46°43'37.94"W	30	2013
SHG	23°45'41.67"S 46°40'6.58"W	28	2012
NBC	23°39'45.18"S 46°39'33.84"W	30	2011

Anhanguera (ANH), Burle Marx (BMX), Piqueri (PQR), Trianon (TRI), Guarapiranga (GRP), Ibirapuera (IBI), Independência (IND), Previdência (PRV), Shangrilá (SHG) and Nabuco (NBC).

Mosquito collections were performed monthly from March 2011 to February 2012 and August 2012 to July 2013. Adult mosquitoes were collected with portable, battery-powered aspirators and CDC CO_2_-baited light traps [51,52]. Mosquitoes were collected over a one-year period in different locations within each urban park. When possible, 30 specimens were selected randomly from each collection site to avoid bias introduced by analyzing siblings (Table 1). The study was approved by the Ethics Committee of the University of São Paulo, and collection permits were provided by the Department of the Environment and Green Areas.

### DNA extraction and PCR reactions

DNA was extracted using the DNEasy Blood and Tissue Kit (Qiagen, Hilden, Germany) following the manufacturer’s protocol. Twelve microsatellite primers labeled with a fluorescent dye (FAM, HEX or NED) and 1μL (~1ng) of purified DNA were used in the PCR reactions, which were performed as described before [41] in an E6331000025 Eppendorf Thermocycler (Mastercycler Nexus Gradient, Eppendorf, Hamburg, Germany) (S1 Table). Amplified fragments were visualized on a 1% agarose gel stained with GelRed Nucleic Acid Gel Stain (Biotium, Hayward, CA, USA) and examined under UV light. PCR products were diluted 1:7 by mixing 3 μL of each product labeled with a different dye with 21 μL of Ultra-Pure Water (Applied Biosystems, Foster City, CA, USA) to a final volume of 30 μL, and 2 μL of the dilution was suspended in 8.925 μL of Hi-Di formamide (Applied Biosystems, Foster City, CA, USA) and 0.075 μL of GeneScan 500 ROX size standard (Applied Biosystems, Foster City, CA, USA) to a final volume of 11 μL. The samples were then sent to the University of São Paulo Center for Human Genome Studies and size-sorted in an ABI 3730 automatic sequencer (Applied Biosystems, Foster City, CA, USA). Fragment analysis was performed using GeneMarker v1.85 (SoftGenetics, State College, PA, USA).

### Genetic analysis

To assess the validity of the microsatellite markers, the number of alleles per locus per population, observed heterozygosity (*H*_*O*_), expected heterozygosity (*H*_*E*_), deviations from Hardy-Weinberg equilibrium (HWE) and inbreeding coefficient (*F*_*IS*_) were calculated with the diveRsity v3.4.0 package in R [53,54]. Linkage disequilibrium was assessed with Genepop v4.2 (http://genepop.curtin.edu.au/) [55], and HP-Rare 1.0 [56] was used to assess allelic richness and private allelic richness in the populations. Multiple tests were corrected using Bonferroni correction method.

Null-allele frequency was assessed for each locus for each population using FreeNA [57]. In order to test if the null alleles influenced the values of genetic heterogeneity (*F*_*ST*_), the same software was used to estimate values of *F*_*ST*_ unbiased by the presence of null alleles. Mantel test was used to compare both *F*_*ST*_ values (biased and unbiased) with ade4 package in R [53,58] using 9,999 permutations.

To compute pairwise values of *F*_*ST*_ (fixation index) and the heterozygosity-based estimator *G”*_*ST*_ for measure population structure of all populations, as proposed by Meirmans and Hedrick [59], the diveRsity package [54] (v3.4.0) in R was used. To assess the gene flow between populations, a genetic network based on a genotype matrix constructed with *F*_*ST*_ values was generated using EDENetworks (v2.18) [60]. Analysis of molecular variance (AMOVA) was performed in Arlequin v3.5.1.2 [61] to detect population differentiation. To test for Isolation by distance, a Mantel test between genetic distance (*F*_*ST*_/(1-*F*_*ST*_)) and geographic distance in kilometers was calculated with ade4 package in R [53,58] using 9,999 permutations.

A Bayesian model-based clustering analysis in STRUCTURE (v2.3.3) was used to infer population structure [62]. Simulations were performed using 100,000 Markov Chain Monte Carlo iterations, with a burn-in period of 100,000 and 10 runs for each value of K (1–10), the analysis was performed assuming Admixture model and correlated allele frequencies between populations. The estimated number of clusters K (ΔK), which identifies genetically homogeneous groups of individuals, was calculated with StructureHarvester (Web v0.6.94) [63].

Discriminant analysis of principal components (DAPC) was used to further assess genetic structure between populations. DAPC was implemented in R using Adegenet package [53,64] for the whole dataset, where groups membership was defined by K = 2 and K = 3. Only populations that were genetically similar were grouped together, which was confirmed using STRUCTURE and pairwise tests for genetic distance (*F*_*ST*_ and *G”*_*ST*_) for all populations. A cross-validation analysis was performed in Adegenet using 30 replicates to determine the number of PCs to be retained in the DAPC, the analysis suggested 60 as the number of PCs associated with the lowest root mean squared error.

## Results

### Marker validation

Hardy-Weinberg equilibrium (HWE) tests conducted for each locus in each population showed *H*_*E*_ (expected heterozygosity) greater than *H*_*O*_ (observed heterozygosity) in 89% of the conducted tests, indicating deviations from the HWE. Average *F*_*IS*_ (inbreeding coefficient) was 0.57072 (S2 Table). After 660 possible comparisons for linkage disequilibrium (LD), 31 tests were statistically significant (*P*<0.05). However, these results could have been produced by chance, as after Bonferroni correction no loci were linked together across the tested populations.

Allelic richness ranged from 1 (NBC) to 13.34 (BMX), and average private allelic richness was low, varying from 0.12 (SHG) to 1.95 (BMX) (S2 Table). Estimated null-allele frequency in the populations was high (>0.40) for locus Di-6 in one population from ten, Tri-18 in three from ten, Tri-20 in five from ten, Tri-41 and Tri-46 in one from ten. The remaining loci yielded a frequency of 0.30 or lower. Locus Tri-44 had a null-allele frequency of zero (S3 Table). There were no significant differences between *F*_*ST*_ values biased and unbiased by the presence of null alleles (r = 0.8698, *P* = 0.001).

### Genetic distance

Pairwise estimates of the fixation index (*F*_*ST*_) and the heterozygosity-based estimator (*G”*_*ST*_) revealed similar results (Table 2). While *F*_*ST*_ values ranged from 0.0047 to 0.1685, indicating that there is some degree of structuring among the populations, *G”*_*ST*_ values were higher (from 0.0525 to 0.446), indicating a high degree of structuring. The lowest *F*_*ST*_ values were observed between samples collected at GRP, IND, SHG, PRV, and IBI and ranged from 0.0047 to 0.0396, suggesting that these populations are very similar to each other and different from the others. The same was observed for *G”*_*ST*_, which ranged from 0.0525 to 0.1494 for the same populations. Although the values of *G”*_*ST*_ were higher than those of *F*_*ST*_, the relationship between *Ae*. *albopictus* populations were similar for both estimators.

**Table 2:** Pairwise *F*_*ST*_ (Weir & Cockerham 1984) estimates (below diagonal) and pairwise *G”*_*ST*_ (Meirmans & Hedrick 2011) estimates (above diagonal) for *Aedes albopictus* populations.

Pop	ANH	BMX	PQR	TRI	GRP	IBI	IND	PRV	SHG	NBC
**ANH**	-	0.1948	0.115	0.2436	0.1954	0.176	0.2006	0.2081	0.1824	0.355
**BMX**	0.042	-	0.1952	0.3552	0.3676	0.3118	0.3403	0.354	0.2912	0.446
**PQR**	0.0222	0.0361	-	0.1469	0.2989	0.2203	0.279	0.282	0.2531	0.3997
**TRI**	0.0535	0.0723	0.0237	-	0.3351	0.2286	0.3142	0.3437	0.2661	0.4397
**GRP**	0.055	0.104	0.0802	0.0931	-	0.1494	0.089	0.124	0.0648	0.2575
**IBI**	0.0426	0.0749	0.0497	0.049	0.0396	-	0.0971	0.0525	0.0694	0.2582
**IND**	0.0536	0.0898	0.0703	0.0805	0.0206	0.0196	-	0.063	0.0579	0.2525
**PRV**	0.0565	0.095	0.072	0.0907	0.0337	0.0047	0.01	-	0.0569	0.2723
**SHG**	0.0464	0.0726	0.0611	0.0633	0.0107	0.009	0.0069	0.0068	-	0.2231
**NBC**	0.134	0.1685	0.1407	0.1689	0.1053	0.0974	0.0992	0.1087	0.0851	-

**Fig 2:**
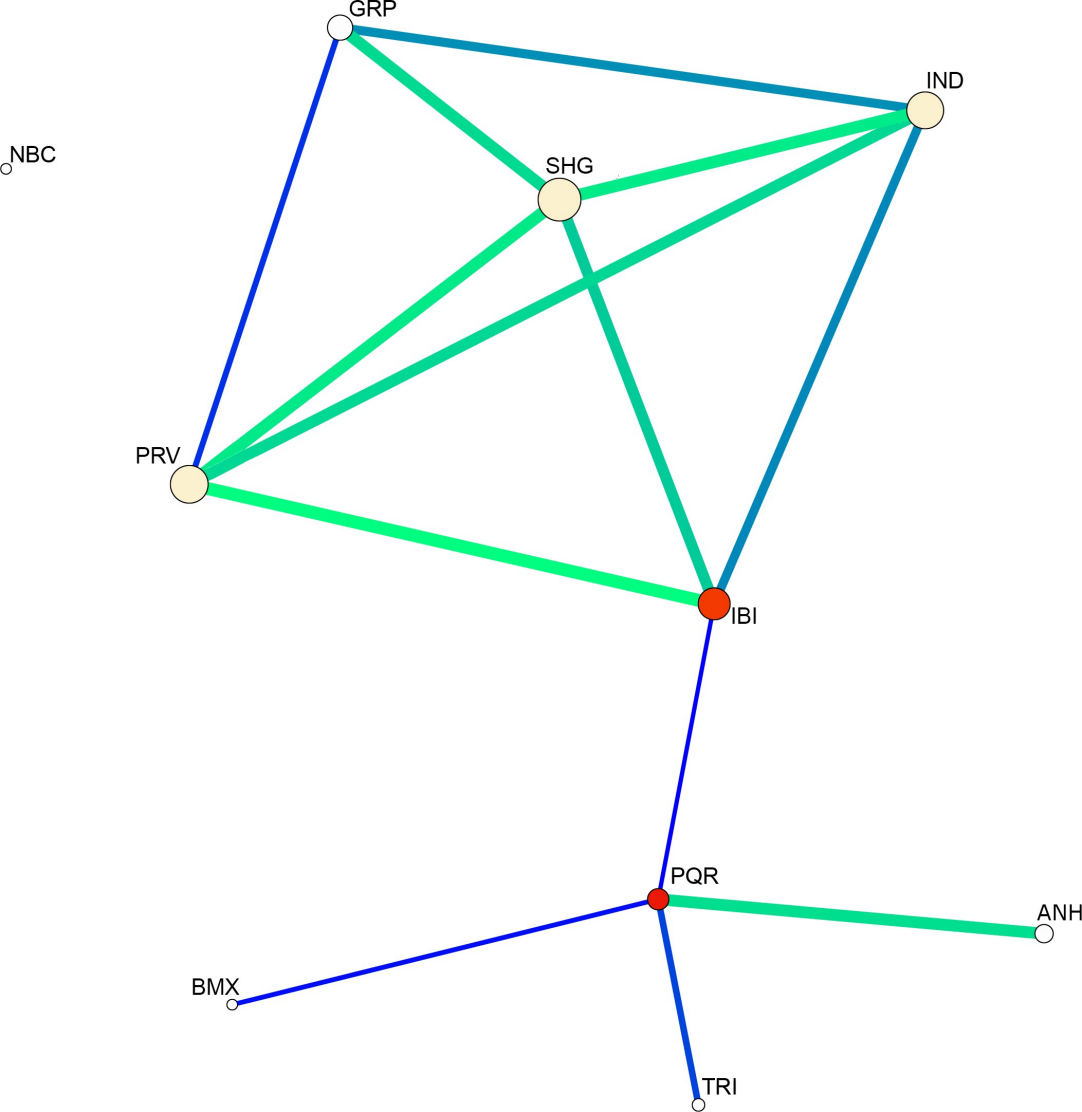
Genetic network based on *F*_*ST*_ values for the *Aedes albopictus* populations in São Paulo studied here. Each node represents a population. The red node represents the populations with most connections. The node size represents the levels of similarity (nearest routes) between populations. The thickness of the lines represents the estimated coancestry coefficient based on *F*_*ST*_ values; the larger the coefficient, the thicker the line.

The genetic network separates the populations into three groups (Fig 2), one composed of GRP, IND, SHG, PRV and IBI, in which significant gene flow occurs between all the populations, and another connecting populations IBI and PQR and composed of ANH, BMX, and TRI, which are connected by PQR. The analysis showed population NBC completely segregated from the others.

AMOVA showed that 90.80% of the variance was estimated to be within populations (S4 Table). Mantel test showed no correlation between genetic distance (*F*_*ST*_/(1-*F*_*ST*_)) and geographic distance (S5 Table), indicating no evidence of isolation by distance (IBD) (r = -0.0262; *P* = 0.5933).

### Bayesian cluster analysis

**Fig 3:**
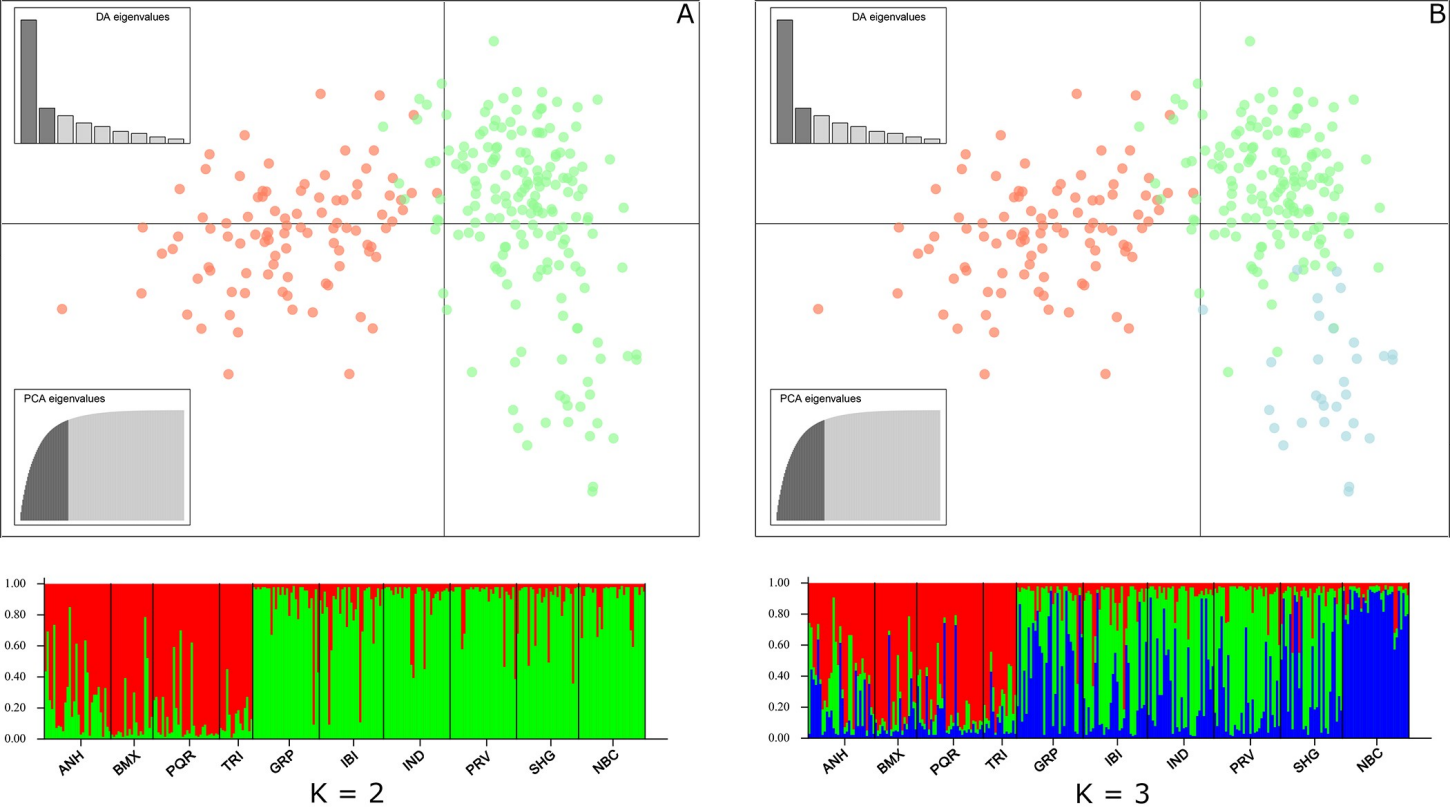
Genetic structure of *Aedes albopictus* populations from São Paulo. Discriminant analysis of the principal components (DAPC) and Bayesian analysis using STRUCTURE for all the *Aedes albopictus* populations, showing the subdivisions for K = 2 (A) and K = 3 (B). Each of the 270 individuals from nine populations is represented by a vertical line divided into different colored segments. The length of each segment represents the probability of the individual belonging to the genetic cluster represented by that color.

The results of the Bayesian cluster analysis and subsequent application of the Evanno method were used to identify the most likely number of genetic groups, which was two according to the ΔK estimator (S1 Fig). The analysis using K = 2 displayed two well defined genetic clusters, one comprising the populations ANH, BMX, PQR, and TRI (red) and the second, comprising GRP, IBI, IND, PRV, SHG, and NBC (green) (Fig 3A). The subsequent analysis using K = 3 resulted in similar results with high genetic similarity within the groups of populations but with the segregation of NBC from the green cluster into a completely independent new genetic cluster (blue) (Fig 3B).

### Multivariate statistical analysis

DAPC explained 94% of the variance in the data, which was capable of partitioning the genetic variation in K = 2 and K = 3 similarly to STRUCTURE analysis (Fig 3). The relationships between populations identified in STRUCTURE analysis and DAPC were consistent with the results obtained with the genetic distance estimators *F*_*ST*_ and *G”*_*ST*_, and the genetic network.

## Discussion

*Aedes albopictus*, a highly anthropophilic species whose dispersal around the world is known to have been mediated by humans [11], has a very close relationship with humans and has high blood-feeding rates in urban areas. Moreover, urban areas can favor faster larval and pupal development of immature specimens of the species [11,65]. Although some studies have suggested that different levels of urbanization may influence the genetic structure of *Ae*. *albopictus* [43,66,67], to date only a handful of studies have addressed this phenomenon at the microscale level [45]. The present study, therefore, investigated the population genetics of *Ae*. *albopictus* at the microscale level, considering the distribution of populations of this species in a city.

Our findings suggest all analyses showing the same genetic structuring pattern between the populations: two distinct groups of populations with limited gene flow between them. Both the *F*_*ST*_ and *G”*_*ST*_ estimators revealed the same relationships between the populations, indicating a high level of structuring between the two groups. DAPC and STRUCTURE analysis revealed high genetic diversity between the two population groups and high similarities within the groups. Furthermore, no evidence of IBD was found for the populations, indicating that the source of genetic variation detected could be other than geographic distance.

Our findings also revealed high levels of homozygosis, high values estimated by the inbreeding coefficient, deviations from the HWE and high molecular variability within the *Ae*. *albopictus* populations, suggesting that the genetic variation between populations probably occurs at a low hierarchical level. Similar results were previously found for native and invasive *Ae*. *albopictus* [8,68], indicating that this is a characteristic of the species and a globally shared pattern [43]. Previous studies have also shown that urbanization and high human population densities may influence the genetic structure of *Ae*. *albopictus* populations [43,66].

The deviations from HWE found in this study were consistent with the pressures *Ae*. *albopictus* populations are probably undergoing in the city of São Paulo. Processes of population expansion following founder effects are particularly important since they are known for leading to deviations in the HWE. Moreover, although the null alleles probabilities for some loci were considered high (>40%), it did not influence the final results, and removing loci with high probabilities did not enhance the results. The presence of null alleles is common in studies using microsatellite markers and are not believed to invalidate the results [47,69,70]. Similar results were also found for *Ae*. *fluviatilis* and *Ae*. *aegypti* collected in the same region [35,36]. Moreover, the high values of inbreeding estimated for the populations studied here agree with other studies [8,68,71]. These values may be explained by the restricted gene flow between populations initially founded by a small number of specimens [43].

Therefore, we believe that there are two hypotheses to explain the pattern of genetic structure in the populations studied here. The first one of the probable causes of the genetic structuring is the limited gene flow between populations due to the low dispersal capability inherent to *Ae*. *albopictus* [43]. A study that screened for SNPs (single nucleotide polymorphisms) in *Ae*. *albopictus* populations worldwide showed the Brazilian populations to be a monophyletic group. The authors suggested that the samples analyzed were derived from a single *Ae*. *albopictus* invasion by a native population from South-East Asia [11]. However, the three Brazilian populations analyzed in the study were from the North and Northeast regions, which are very far from the areas where the populations analyzed here were sampled.

Previous studies have suggested that the dispersal pattern of *Ae*. *albopictus* populations may have been chaotic and that this may have played a role in maintaining genetic diversity in invasive populations around the world [72]. Our second hypothesis is therefore that multiple introductions of *Ae*. *albopictus* probably occurred. Under this hypothesis, the population structure found in this study is due to multiple introductions of *Ae*. *albopictus* in the city of São Paulo, leading to different dispersal patterns within the city and more genetic heterogeneity. Manni et al. [72], postulated that multiple introductions could increase genetic diversity, favoring expansion and adaptation. The restriction on gene flow between the population groups observed here may also mean that more than one introduction occurred, as the city was already vast in 1986 when this species was first introduced and its topography may have acted as a barrier to gene flow.

There are several mechanisms able to increase or decrease the genetic variation in a given population [73]. Invading and colonizing new areas may have distinct outcomes due to environmental heterogeneity and how local resources can be explored by the invasive species [43]. Moreover, human behavior can actively drive the dispersal of *Ae*. *albopictus*, substantially impacting its population dynamics and genetic diversity patterns [74]. At this point, there is no scientific consensus on the outcome of population expansion in the genetic structuring of *Ae*. *albopictus*. In our opinion, the two hypotheses suggested here could be explored by future studies by focusing on broader geographic scales and also employing longitudinal experimental designs. This approach would make it possible to identify trends of founder effect and population expansion, and movement and subsequent colonization of new areas by *Ae*. *albopictus*.

Previous studies suggested that *Ae*. *albopictus* populations have reduced gene flow between densely urbanized areas and rural areas [8,45]. A similar phenomenon was observed in the invasive mosquito *Ae*. *aegypti* in the city of São Paulo [36]. However, a positive association between a decrease in genetic structure and an increase in urbanization was found for species native to Brazil, such as *Ae*. *fluviatilis* and *Cx*. *nigripalpus* [35,38]. This contrast between the genetic structure of native and invasive species may indicate that invasive species are better able to adapt and thrive in urban environments, an advantage that is clearly of epidemiological relevance.

Although the invasive mosquito *Ae*. *aegypti* had been eradicated from Brazil, it reinfested the country in the 1970s [75,76]. The species rapidly recolonized the whole country, displaying genetic structuring even on a microgeographic scale [36,77], and is now the primary vector of ZIKV, CHIKV, and DENV in Brazil [78]. The same phenomenon is likely to have occurred with *Ae*. *albopictus* as this mosquito was first recorded in Brazil in 1986 and approximately 30 years later had spread to 24 of the 27 Brazilian states [20].

*Aedes albopictus*, which is widespread in Brazil, probably entered the country through the port of Espírito Santo and was then passively dispersed in cargo transported on highways, as it was first reported on the Rio-São Paulo highway in 1986 [12,22]. Although the species is found in sylvatic and peri-urban areas in Brazil, its distribution is closely associated with the presence of humans, and it can move quickly between sylvatic and urban environments [7,24]. The ability of *Ae*. *albopictus* to invade new areas and expand explains why these mosquito populations appear to be well established and thriving in the city of São Paulo. Human movements throughout the city and the lack of large-scale mosquito control measures in Brazil have probably favored the dispersal and establishment of this species in the city. These factors, together with the epidemiological importance of this mosquito, highlight the need for further studies of the population genetics of this species in Brazil focusing on broader geographic scales and longitudinal experimental designs. *Aedes albopictus* populations are genetically diverse and can diverge in their ecology and behavior, affecting their resistance to insecticides and vector capacity, among other factors.

## Supporting information

S1 Fig Graph of ΔK showing K = 2 as the most probable number of genetic groups for the *Aedes albopictus* populations in São Paulo studied here. (TIF)

S1 Table Microsatellite loci amplified in *Aedes albopictus*. (DOCX)

S2 Table Characterization of the 12 loci analyzed in 10 *Aedes albopictus* populations. N (Number of individuals), A (Number of alleles), Ar (Allele richness), Pr (Private allele richness), *H*_*O*_ (Observed heterozygosity), *H*_*E*_ (Expected heterozygosity), HWE (Hardy-Weinberg Equilibrium) and *F*_*IS*_ (Inbreeding Coefficient).
(DOCX)

S3 Table Estimation of null allele frequencies per locus per population of *Aedes albopictus* from Sao Paulo, Brazil. (DOCX)

S4 Table Global AMOVA results based on 12 variable loci in the *Aedes albopictus* populations in São Paulo, Brazil. (DOCX)

S5 Table Below the diagonal: Genetic distance (*F*_*ST*_/(1-*F*_*ST*_)) for all 10 populations of *Aedes albopictus* from São Paulo. Above the diagonal: geographic distances between populations in kilometers.
(DOCX)
