# Supplementary figures and images for: Population structuring of the invasive mosquito Aedes albopictus (Diptera: Culicidae) on a microgeographic scale
Source: PLoS One. 2019 Aug 2;14(8):e0220773. doi: 10.1371/journal.pone.0220773 (PMC6677317; doi:10.1371/journal.pone.0220773)

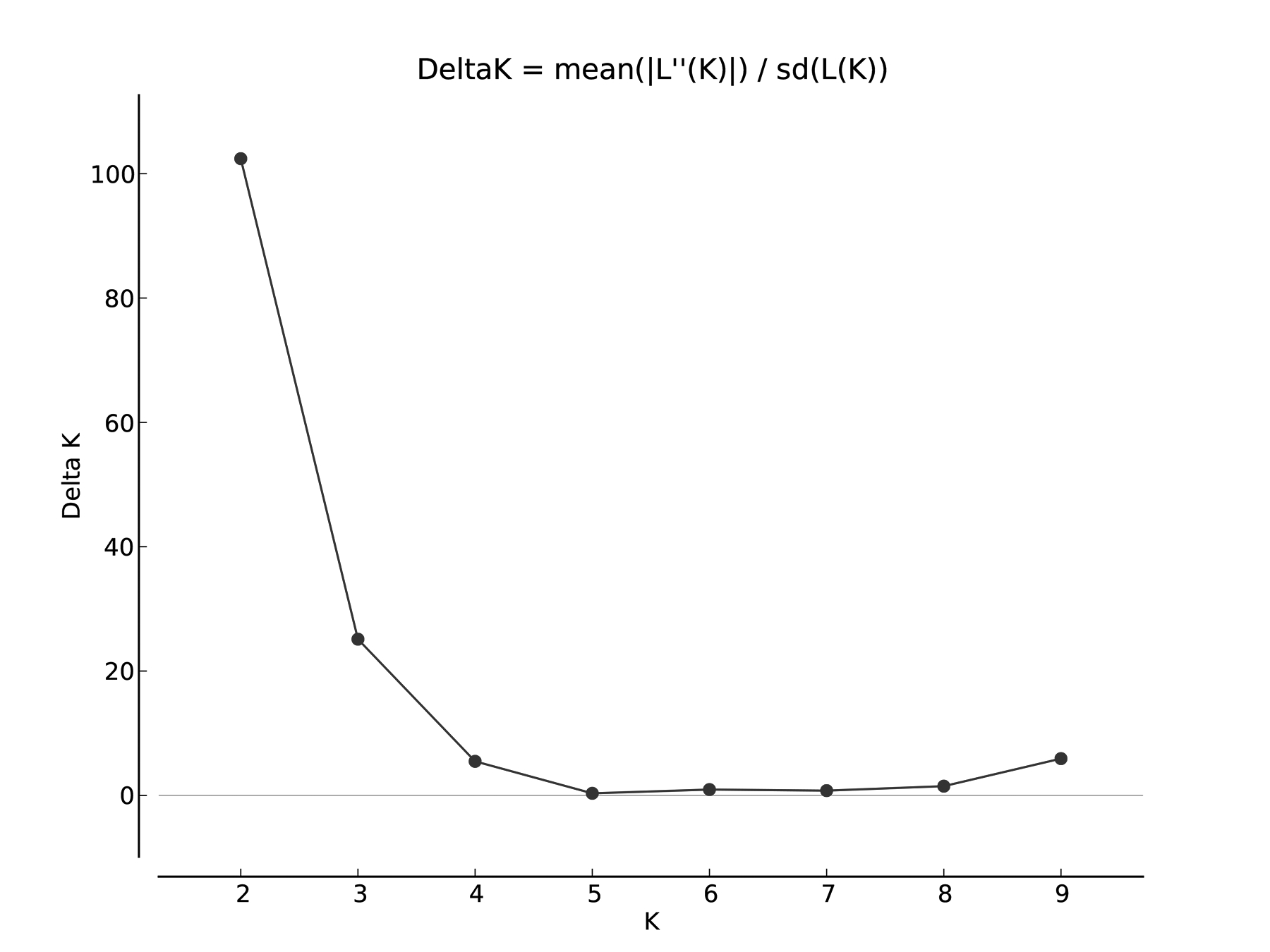

Supplement: S1 Fig — (TIF) [file pone.0220773.s001.tif]
