# Supplementary material for: Population structuring of the invasive mosquito Aedes albopictus (Diptera: Culicidae) on a microgeographic scale
Source: PLoS One. 2019 Aug 2;14(8):e0220773. doi: 10.1371/journal.pone.0220773 (PMC6677317; doi:10.1371/journal.pone.0220773)
Supplement: S1 Table — (DOCX) [file pone.0220773.s002.docx]

S1 Table. Microsatellite loci amplified in *Aedes albopictus*.

| Primer | Fluorescent Dye | Sequence | Reference |
| --- | --- | --- | --- |
| Alb-di-4 | 6'FAM | F: TGGCGACCTATTATACCCGC | Beebe et al.[41] |
|  |  | R: CAACTCGTTCCTTGACCGTG |  |
| Alb-di-6 | HEX | F: TCTTCATCTACGCTGTGCTC | Beebe et al.[41] |
|  |  | R: GACGCCAATCCGACAAAGTC |  |
| Alb-tri-3 | NED | F: AGATGTGTCGCAATGCTTCC | Beebe et al.[41] |
|  |  | R: GATTCGGTGATGTTGAGGCC |  |
| Alb-tri-6 | 6'FAM | F: AGCACGAGTACAGAATGTGC | Beebe et al.[41] |
|  |  | R: TGGCCTCCTACCGTTTATCTG |  |
| Alb-tri-18 | HEX | F: ACACAATTGCCGTTCAGCTC | Beebe et al.[41] |
|  |  | R: CGTCTAATAGCTCCGGTCCC |  |
| Alb-tri-20 | NED | F: GTGCCGTTGATCATCCTGTC | Beebe et al.[41] |
|  |  | R: TCCAGCACCGTGAGTAATCC |  |
| Alb-tri-25 | 6'FAM | F: CCAACCAACAACCCAGGAAC | Beebe et al.[41] |
|  |  | R: TACGATGCGCAACCATCATC |  |
| Alb-tri-33 | HEX | F: GGCTGCTGTTGTTGGTACG | Beebe et al.[41] |
|  |  | R: CACGTTCAATCACCGGTTCC |  |
| Alb-tri-41 | NED | F: GATCGATTTGGGAGCTTCTG | Beebe et al.[41] |
|  |  | R: GAACCTCTTCTCGCTTGGCT |  |
| Alb-tri-44 | 6'FAM | F: CACTCGCGCGTGTTCTTC | Beebe et al.[41] |
|  |  | R: GACGCACCATCAGCATCATC |  |
| Alb-tri-45 | HEX | F: TTTCAGCTCGGTGTTATGGC | Beebe et al.[41] |
|  |  | R: TGATGTTGATGATGATGACTACGA |  |
| Alb-tri-46 | NED | F: TTCACAACATACGGAATCGC | Beebe et al.[41] |
|  |  | R: GGTCCGGTGTAATAGCCTCC |  |
