# Supplementary material for: Population structuring of the invasive mosquito Aedes albopictus (Diptera: Culicidae) on a microgeographic scale
Source: PLoS One. 2019 Aug 2;14(8):e0220773. doi: 10.1371/journal.pone.0220773 (PMC6677317; doi:10.1371/journal.pone.0220773)
Supplement: S2 Table — N (Number of individuals), A (Number of alleles), Ar (Allele richness), Pr (Private allele richness), HO (Observed heterozygosity), HE (Expected heterozygosity), HWE (Hardy-Weinberg Equilibrium) and FIS (Inbreeding Coefficient). (DOCX) [file pone.0220773.s003.docx]

S2 Table. Characterization of the 12 loci analyzed in 10 *Aedes albopictus* populations.

| ANG | Di-4 | Di-6 | Tri-3 | Tri-6 | Tri-18 | Tri20 | Tri-25 | Tri-33 | Tri-41 | Tri-44 | Tri-45 | Tri-46 |
| --- | --- | --- | --- | --- | --- | --- | --- | --- | --- | --- | --- | --- |
| N | 30 | 30 | 30 | 28 | 30 | 30 | 30 | 30 | 30 | 30 | 30 | 30 |
| A | 5 | 10 | 7 | 15 | 8 | 15 | 6 | 7 | 7 | 8 | 13 | 12 |
| Ar | 3.3998 | 7.9823 | 5.1198 | 11.8612 | 7.1998 | 10.0581 | 5.3571 | 5.5481 | 6.3869 | 6.2904 | 9.9844 | 7.6674 |
| Pr | 0.7832 | 1.1896 | 0.3351 | 3.8317 | 0.1308 | 2.7526 | 0.0013 | 0.0567 | 0.0003 | 0.8725 | 1.6955 | 0.8566 |
| Ho | 0.1 | 0.9 | 0.73 | 0.25 | 0.33 | 0.6 | 0.3 | 0.1 | 0.47 | 0.97 | 0.5 | 0.57 |
| He | 0.4 | 0.8 | 0.72 | 0.9 | 0.8 | 0.83 | 0.66 | 0.51 | 0.8 | 0.7 | 0.87 | 0.63 |
| HWE | 0.001 | 0.002 | 0.035 | 0.001 | 0.001 | 0.001 | 0.001 | 0.001 | 0.001 | 0.001 | 0.001 | 0.001 |
| Fis | 0.7479 | -0.118 | -0.0248 | 0.7222 | 0.581 | 0.2795 | 0.5443 | 0.8028 | 0.4187 | -0.3831 | 0.426 | 0.0949 |
| BMX | Di-4 | di-6 | Tri-3 | Tri-6 | Tri-18 | Tri20 | Tri-25 | Tri-33 | Tri-41 | Tri-44 | Tri-45 | Tri-46 |
| N | 19 | 19 | 19 | 19 | 19 | 19 | 19 | 19 | 19 | 19 | 19 | 19 |
| A | 2 | 8 | 3 | 15 | 13 | 14 | 7 | 8 | 13 | 9 | 10 | 9 |
| Ar | 2 | 7.6556 | 3 | 13.3437 | 11.6215 | 12.2447 | 6.5301 | 7.0768 | 11.1351 | 7.9545 | 9.264 | 7.8686 |
| Pr | 0 | 1.0277 | 0 | 4.5142 | 5.2179 | 5.0234 | 1.0731 | 0.7396 | 2.6653 | 0.96 | 1.3954 | 0.7468 |
| Ho | 0 | 0.84 | 0.63 | 0.32 | 0.11 | 0.58 | 0.21 | 0.16 | 0.84 | 1 | 0.37 | 0.32 |
| He | 0.43 | 0.84 | 0.65 | 0.9 | 0.87 | 0.87 | 0.6 | 0.6 | 0.86 | 0.73 | 0.83 | 0.72 |
| HWE | - | 0.113 | 0.044 | 0.001 | 0.001 | 0.001 | 0.001 | 0.001 | 0.001 | 0.032 | 0.001 | 0.001 |
| Fis | 1 | -0.0033 | 0.0339 | 0.6503 | 0.8786 | 0.3312 | 0.6498 | 0.7385 | 0.0225 | -0.3779 | 0.5567 | 0.5624 |
| GRP | Di-4 | di-6 | Tri-3 | Tri-6 | Tri-18 | Tri20 | Tri-25 | Tri-33 | Tri-41 | Tri-44 | Tri-45 | Tri-46 |
| N | 30 | 30 | 29 | 29 | 29 | 30 | 30 | 29 | 30 | 29 | 29 | 29 |
| A | 2 | 6 | 3 | 5 | 6 | 7 | 8 | 7 | 6 | 5 | 9 | 7 |
| Ar | 1.9263 | 5.1769 | 2.4828 | 4.4179 | 5.6399 | 5.8838 | 5.8354 | 5.6631 | 5.0946 | 4.2195 | 6.8818 | 6.2129 |
| Pr | 0.9263 | 0.0074 | 0.4828 | 0.0003 | 0 | 0.4667 | 1.4202 | 0.2551 | 0 | 0.0043 | 0.49 | 0.0118 |
| Ho | 0 | 0.03 | 0.03 | 0.34 | 0 | 0.03 | 0.17 | 0.45 | 0.1 | 1 | 0.31 | 0.21 |
| He | 0.12 | 0.73 | 0.52 | 0.69 | 0.65 | 0.7 | 0.66 | 0.63 | 0.65 | 0.65 | 0.71 | 0.7 |
| HWE | - | 0.001 | 0.001 | 0.001 | - | 0.001 | 0.001 | 0.001 | 0.001 | 0.034 | 0.001 | 0.001 |
| Fis | 1 | 0.9541 | 0.9331 | 0.5013 | 1 | 0.9521 | 0.7466 | 0.2867 | 0.8452 | -0.5445 | 0.5643 | 0.7058 |
| IBI | Di-4 | di-6 | Tri-3 | Tri-6 | Tri-18 | Tri20 | Tri-25 | Tri-33 | Tri-41 | Tri-44 | Tri-45 | Tri-46 |
| N | 29 | 29 | 29 | 28 | 29 | 29 | 29 | 29 | 29 | 29 | 29 | 29 |
| A | 2 | 6 | 3 | 9 | 8 | 7 | 5 | 7 | 9 | 6 | 10 | 6 |
| Ar | 1.9969 | 4.9533 | 2.9853 | 7.6861 | 7.6337 | 6.6543 | 4.4175 | 5.369 | 7.307 | 4.9495 | 8.2697 | 5.409 |
| Pr | 0 | 0.7368 | 0.0053 | 0.0593 | 0.0142 | 0.1101 | 0.0005 | 0.2523 | 1.7201 | 0.7402 | 0.7441 | 0.9433 |
| Ho | 0 | 0.1 | 0 | 0.21 | 0 | 0 | 0.03 | 0.1 | 0.03 | 1 | 0.28 | 0.03 |
| He | 0.24 | 0.61 | 0.57 | 0.75 | 0.84 | 0.82 | 0.64 | 0.64 | 0.78 | 0.69 | 0.76 | 0.71 |
| HWE | - | 0.001 | - | 0.001 | - | - | 0.001 | 0.001 | 0.001 | 0.001 | 0.001 | 0.001 |
| Fis | 1 | 0.8309 | 1 | 0.7138 | 1 | 1 | 0.9458 | 0.8383 | 0.9556 | -0.45 | 0.6389 | 0.9517 |
| IND | Di-4 | di-6 | Tri-3 | Tri-6 | Tri-18 | Tri20 | Tri-25 | Tri-33 | Tri-41 | Tri-44 | Tri-45 | Tri-46 |
| N | 30 | 30 | 30 | 30 | 30 | 30 | 30 | 30 | 30 | 30 | 30 | 30 |
| A | 2 | 7 | 3 | 11 | 8 | 7 | 4 | 5 | 4 | 5 | 8 | 7 |
| Ar | 1.9819 | 5.8234 | 2.7198 | 9.0187 | 6.5522 | 6.6229 | 3.7017 | 4.5688 | 3.4666 | 4.709 | 6.7859 | 5.9012 |
| Pr | 0 | 0.1592 | 0.0002 | 0.946 | 1.1167 | 0.7198 | 0 | 0 | 0 | 0 | 0.4819 | 0.0012 |
| Ho | 0 | 0.03 | 0 | 0.2 | 0.07 | 0 | 0.43 | 0.1 | 0.03 | 1 | 0.23 | 0.13 |
| He | 0.18 | 0.72 | 0.5 | 0.82 | 0.77 | 0.81 | 0.57 | 0.56 | 0.64 | 0.67 | 0.72 | 0.75 |
| HWE | - | 0.001 | - | 0.001 | 0.001 | - | 0.051 | 0.001 | 0.001 | 0.039 | 0.001 | 0.001 |
| Fis | 1 | 0.9535 | 1 | 0.7548 | 0.9131 | 1 | 0.2375 | 0.8198 | 0.948 | -0.4864 | 0.6747 | 0.8224 |
| NBC | Di-4 | di-6 | Tri-3 | Tri-6 | Tri-18 | Tri20 | Tri-25 | Tri-33 | Tri-41 | Tri-44 | Tri-45 | Tri-46 |
| N | 30 | 30 | 30 | 30 | 30 | 30 | 30 | 30 | 30 | 30 | 30 | 30 |
| A | 3 | 6 | 3 | 4 | 6 | 5 | 1 | 5 | 4 | 5 | 4 | 6 |
| Ar | 2.9082 | 4.3732 | 2.9263 | 3.646 | 4.6309 | 4.5723 | 1 | 3.8881 | 3.1864 | 4.3213 | 3.646 | 5.4114 |
| Pr | 0 | 0.4667 | 0 | 0.0495 | 0.3797 | 0 | 0 | 0.7204 | 0 | 0 | 0.7198 | 0.1308 |
| Ho | 0 | 0.1 | 0.07 | 0 | 0.07 | 0 | 0 | 0.03 | 0.03 | 1 | 0.13 | 0.43 |
| He | 0.29 | 0.44 | 0.52 | 0.52 | 0.48 | 0.62 | 0 | 0.32 | 0.46 | 0.65 | 0.58 | 0.74 |
| HWE | - | 0.001 | 0.001 | - | 0.001 | - | - | 0.001 | 0.001 | 0.001 | 0.001 | 0.001 |
| Fis | 1 | 0.7716 | 0.8729 | 1 | 0.8613 | 1 | - | 0.896 | 0.9276 | -0.5358 | 0.7703 | 0.4166 |
| PQR | Di-4 | di-6 | Tri-3 | Tri-6 | Tri-18 | Tri20 | Tri-25 | Tri-33 | Tri-41 | Tri-44 | Tri-45 | Tri-46 |
| N | 30 | 29 | 30 | 30 | 30 | 30 | 30 | 30 | 30 | 30 | 30 | 30 |
| A | 6 | 11 | 7 | 14 | 13 | 17 | 7 | 8 | 12 | 6 | 9 | 13 |
| Ar | 5.0406 | 8.4625 | 5.7146 | 10.0921 | 10.617 | 12.6933 | 5.8859 | 6.4785 | 8.9801 | 5.1497 | 7.7493 | 9.9605 |
| Pr | 1.4247 | 1.6139 | 1.4351 | 1.992 | 2.3606 | 3.0157 | 0.2881 | 0.0591 | 1.0695 | 0.0119 | 0.3841 | 1.5388 |
| Ho | 0.03 | 0.41 | 0.2 | 0.6 | 0.3 | 0.83 | 0.27 | 0.13 | 0.77 | 1 | 0.77 | 0.9 |
| He | 0.69 | 0.81 | 0.74 | 0.82 | 0.86 | 0.9 | 0.62 | 0.7 | 0.84 | 0.63 | 0.82 | 0.82 |
| HWE | 0.001 | 0.001 | 0.001 | 0.001 | 0.001 | 0.024 | 0.001 | 0.001 | 0.001 | 0.197 | 0.524 | 0.029 |
| Fis | 0.9517 | 0.4897 | 0.7307 | 0.2663 | 0.6532 | 0.0775 | 0.568 | 0.8103 | 0.0879 | -0.5831 | 0.0625 | -0.0961 |
| PRV | Di-4 | di-6 | Tri-3 | Tri-6 | Tri-18 | Tri20 | Tri-25 | Tri-33 | Tri-41 | Tri-44 | Tri-45 | Tri-46 |
| N | 29 | 30 | 30 | 30 | 30 | 30 | 30 | 30 | 30 | 30 | 30 | 30 |
| A | 2 | 8 | 4 | 8 | 9 | 6 | 5 | 4 | 3 | 6 | 7 | 5 |
| Ar | 1.9969 | 6.0877 | 3.1864 | 7.1285 | 7.7819 | 5.5682 | 4.7114 | 3.7189 | 2.9998 | 4.9236 | 6.4599 | 4.4387 |
| Pr | 0 | 0.4667 | 0.0186 | 0.0021 | 0.9687 | 0 | 0 | 0.0565 | 0 | 0.2414 | 0.0004 | 0.1894 |
| Ho | 0 | 0.2 | 0.03 | 0.37 | 0.1 | 0 | 0.27 | 0 | 0 | 1 | 0.27 | 0 |
| He | 0.24 | 0.63 | 0.54 | 0.77 | 0.8 | 0.71 | 0.67 | 0.6 | 0.6 | 0.68 | 0.74 | 0.65 |
| HWE | - | 0.002 | 0.001 | 0.001 | 0.001 | - | 0.001 | - | - | 0.001 | 0.001 | - |
| Fis | 1 | 0.6823 | 0.9378 | 0.5224 | 0.8757 | 1 | 0.604 | 1 | 1 | -0.461 | 0.6394 | 1 |
| SHG | Di-4 | di-6 | Tri-3 | Tri-6 | Tri-18 | Tri20 | Tri-25 | Tri-33 | Tri-41 | Tri-44 | Tri-45 | Tri-46 |
| N | 28 | 28 | 28 | 28 | 28 | 28 | 28 | 28 | 28 | 28 | 27 | 28 |
| A | 4 | 7 | 4 | 9 | 7 | 6 | 7 | 5 | 5 | 6 | 7 | 5 |
| Ar | 3.0091 | 6.0078 | 3.6364 | 7.594 | 6.2078 | 5.9189 | 5.4185 | 4.1988 | 4.4969 | 4.9873 | 5.9128 | 4.9858 |
| Pr | 0 | 0.0002 | 0.0002 | 0.2115 | 0.0156 | 0 | 1.0532 | 0.0007 | 0.1468 | 0.0046 | 0.0029 | 0.0001 |
| Ho | 0.04 | 0.11 | 0.11 | 0.21 | 0 | 0 | 0.25 | 0.04 | 0.04 | 1 | 0.07 | 0 |
| He | 0.17 | 0.77 | 0.58 | 0.77 | 0.74 | 0.79 | 0.63 | 0.6 | 0.71 | 0.67 | 0.59 | 0.77 |
| HWE | 0.005 | 0.001 | 0.001 | 0.001 | - | - | 0.001 | 0.001 | 0.001 | 0.001 | 0.001 | - |
| Fis | 0.7871 | 0.8609 | 0.8144 | 0.7216 | 1 | 1 | 0.6004 | 0.9402 | 0.9495 | -0.4848 | 0.8753 | 1 |
| TRI | Di-4 | di-6 | Tri-3 | Tri-6 | Tri-18 | Tri20 | Tri-25 | Tri-33 | Tri-41 | Tri-44 | Tri-45 | Tri-46 |
| N | 15 | 15 | 15 | 14 | 15 | 15 | 15 | 15 | 15 | 15 | 15 | 15 |
| A | 3 | 10 | 3 | 8 | 8 | 10 | 6 | 8 | 11 | 6 | 5 | 10 |
| Ar | 3 | 9.6621 | 3 | 8 | 7.731 | 9.7931 | 5.9954 | 7.9885 | 10.7241 | 5.8644 | 4.9333 | 9.6621 |
| Pr | 0.5 | 3.383 | 0 | 0.0688 | 1.1067 | 0.5366 | 0.9978 | 2.0009 | 2.9424 | 0.2632 | 1 | 2.525 |
| Ho | 0.2 | 0.67 | 0.4 | 0.21 | 0.33 | 0.67 | 0.2 | 0.27 | 0.8 | 0.87 | 0.4 | 0.33 |
| He | 0.46 | 0.78 | 0.6 | 0.82 | 0.75 | 0.84 | 0.77 | 0.81 | 0.85 | 0.68 | 0.74 | 0.74 |
| HWE | 0.025 | 0.032 | 0.141 | 0.001 | 0.001 | 0.078 | 0.001 | 0.001 | 0.001 | 0.043 | 0.001 | 0.001 |
| Fis | 0.5694 | 0.1453 | 0.3382 | 0.7375 | 0.5549 | 0.2042 | 0.7406 | 0.6703 | 0.0576 | -0.2662 | 0.4595 | 0.5495 |

N (Number of individuals), A (Number of alleles), Ar (Allele richness), Pr (Private allele richness), *H_O_* (Observed heterozygosity), *H_E_* (Expected heterozygosity), HWE (Hardy-Weinberg Equilibrium) and *F_IS_* (Inbreeding Coefficient).
