# Supplementary material for: Population structuring of the invasive mosquito Aedes albopictus (Diptera: Culicidae) on a microgeographic scale
Source: PLoS One. 2019 Aug 2;14(8):e0220773. doi: 10.1371/journal.pone.0220773 (PMC6677317; doi:10.1371/journal.pone.0220773)
Supplement: S3 Table — (DOCX) [file pone.0220773.s004.docx]

S3 Table. Estimation of null allele frequencies per locus per population of *Aedes albopictus* from Sao Paulo, Brazil.

| **Locus** | **Population** | **Estimate of null allele frequency** | **Locus** | **Population** | **Estimate of null allele frequency** | **Locus** | **Population** | **Estimate of null allele frequency** |
| --- | --- | --- | --- | --- | --- | --- | --- | --- |
| Di-4 | ANH | 0.23290 | Di-6 | ANH | 0.01437 | Tri-3 | ANH | 0.00001 |
|  | BMX | 0.31078 |  | BMX | 0.03961 |  | BMX | 0.00573 |
|  | PQR | 0.38934 |  | PQR | 0.21352 |  | PQR | 0.31153 |
|  | TRI | 0.17611 |  | TRI | 0.02453 |  | TRI | 0.13126 |
|  | GRP | 0.17030 |  | GRP | 0.40183 |  | GRP | 0.31547 |
|  | IBI | 0.23306 |  | IBI | 0.32417 |  | IBI | 0.36523 |
|  | IND | 0.20369 |  | IND | 0.39898 |  | IND | 0.33981 |
|  | PRV | 0.23306 |  | PRV | 0.26936 |  | PRV | 0.32913 |
|  | SHG | 0.17009 |  | SHG | 0.37442 |  | SHG | 0.29648 |
|  | NBC | 0.26390 |  | NBC | 0.25413 |  | NBC | 0.30050 |
| Tri-6 | ANH | 0.34210 | Tri-18 | ANH | 0.26233 | Tri-20 | ANH | 0.10217 |
|  | BMX | 0.31082 |  | BMX | 0.40812 |  | BMX | 0.14538 |
|  | PQR | 0.11234 |  | PQR | 0.30316 |  | PQR | 0.04967 |
|  | TRI | 0.33507 |  | TRI | 0.22994 |  | TRI | 0.05422 |
|  | GRP | 0.20604 |  | GRP | 0.39960 |  | GRP | 0.39456 |
|  | IBI | 0.30859 |  | IBI | 0.45662 |  | IBI | 0.45085 |
|  | IND | 0.34190 |  | IND | 0.39631 |  | IND | 0.44878 |
|  | PRV | 0.22785 |  | PRV | 0.39098 |  | PRV | 0.41820 |
|  | SHG | 0.31561 |  | SHG | 0.42807 |  | SHG | 0.44052 |
|  | NBC | 0.35222 |  | NBC | 0.29955 |  | NBC | 0.38532 |
| Tri-25 | ANH | 0.21938 | Tri-33 | ANH | 0.29082 | Tri-41 | ANH | 0.19105 |
|  | BMX | 0.25819 |  | BMX | 0.29434 |  | BMX | 0.00287 |
|  | PQR | 0.20461 |  | PQR | 0.33186 |  | PQR | 0.04841 |
|  | TRI | 0.32460 |  | TRI | 0.30781 |  | TRI | 0.05017 |
|  | GRP | 0.29332 |  | GRP | 0.12915 |  | GRP | 0.33731 |
|  | IBI | 0.37309 |  | IBI | 0.33366 |  | IBI | 0.41778 |
|  | IND | 0.09524 |  | IND | 0.30378 |  | IND | 0.37212 |
|  | PRV | 0.25205 |  | PRV | 0.37877 |  | PRV | 0.38016 |
|  | SHG | 0.23494 |  | SHG | 0.35485 |  | SHG | 0.39403 |
|  | NBC | 0.00100 |  | NBC | 0.25483 |  | NBC | 0.30471 |
| Tri-44 | ANH | 0.00018 | Tri-45 | ANH | 0.19780 | Tri-46 | ANH | 0.01053 |
|  | BMX | 0.00000 |  | BMX | 0.25544 |  | BMX | 0.22786 |
|  | PQR | 0.00000 |  | PQR | 0.03895 |  | PQR | 0.00001 |
|  | TRI | 0.00000 |  | TRI | 0.20076 |  | TRI | 0.24336 |
|  | GRP | 0.00000 |  | GRP | 0.23313 |  | GRP | 0.29391 |
|  | IBI | 0.00000 |  | IBI | 0.27461 |  | IBI | 0.39889 |
|  | IND | 0.00000 |  | IND | 0.28157 |  | IND | 0.35181 |
|  | PRV | 0.00000 |  | PRV | 0.27286 |  | PRV | 0.39661 |
|  | SHG | 0.00000 |  | SHG | 0.33553 |  | SHG | 0.43556 |
|  | NBC | 0.00000 |  | NBC | 0.27961 |  | NBC | 0.19789 |
