# Supplementary material for: Population structuring of the invasive mosquito Aedes albopictus (Diptera: Culicidae) on a microgeographic scale
Source: PLoS One. 2019 Aug 2;14(8):e0220773. doi: 10.1371/journal.pone.0220773 (PMC6677317; doi:10.1371/journal.pone.0220773)
Supplement: S4 Table — (DOCX) [file pone.0220773.s005.docx]

S4 Table. Global AMOVA results based on 12 variable loci in the *Aedes albopictus* populations in São Paulo, Brazil.

| Source of variation | Degrees of freedom | Sum of squares | Variance components | Percentage variation | *P*-value |
| --- | --- | --- | --- | --- | --- |
| Between the groups | 2 | 101.825 | 0.24591 Va | 5.60% | <0.0001 |
| Between the populations within the groups | 7 | 85.209 | 0.15386 Vb | 3.51% | <0.0001 |
| Within populations | 532 | 2.121.407 | 3.98761 Vc | 90.89% | <0.0001 |
