# Supplementary material for: Population structuring of the invasive mosquito Aedes albopictus (Diptera: Culicidae) on a microgeographic scale
Source: PLoS One. 2019 Aug 2;14(8):e0220773. doi: 10.1371/journal.pone.0220773 (PMC6677317; doi:10.1371/journal.pone.0220773)
Supplement: S5 Table — Above the diagonal: geographic distances between populations in kilometers. (DOCX) [file pone.0220773.s006.docx]

S5 Table. Below the diagonal: Genetic distance (*F_ST_*/(1-*F_ST_*)) for all 10 populations of *Aedes albopictus* from São Paulo. Above the diagonal: geographic distances between populations in kilometers.

| Pop | ANH | BMX | PQR | TRI | GRP | IBI | IND | PRV | SHG | NBC |
| --- | --- | --- | --- | --- | --- | --- | --- | --- | --- | --- |
| ANH | - | 24.3 | 24.4 | 20.1 | 28.7 | 22.5 | 25.1 | 18.5 | 39.5 | 29.6 |
| BMX | 0.042 | - | 18.8 | 10.1 | 5 | 8.1 | 12.5 | 5.6 | 15.6 | 7.2 |
| PQR | 0.0222 | 0.0361 | - | 9.33 | 23 | 10.8 | 7.3 | 16.7 | 27.8 | 17.3 |
| TRI | 0.0535 | 0.0723 | 0.0237 | - | 12.6 | 2.8 | 5.5 | 7.4 | 22.3 | 11.2 |
| GRP | 0.055 | 0.104 | 0.0802 | 0.0931 | - | 12.6 | 16.2 | 10.6 | 11.6 | 7.7 |
| IBI | 0.0426 | 0.0749 | 0.0497 | 0.049 | 0.0396 | - | 4.9 | 7.15 | 19.5 | 8.35 |
| IND | 0.0536 | 0.0898 | 0.0703 | 0.0805 | 0.0206 | 0.0196 | - | 12 | 20.8 | 10.1 |
| PRV | 0.0565 | 0.095 | 0.072 | 0.0907 | 0.0337 | 0.0047 | 0.01 | - | 21 | 11.5 |
| SHG | 0.0464 | 0.0726 | 0.0611 | 0.0633 | 0.0107 | 0.009 | 0.0069 | 0.0068 | - | 11 |
| NBC | 0.134 | 0.1685 | 0.1407 | 0.1689 | 0.1053 | 0.0974 | 0.0992 | 0.1087 | 0.0851 | - |
